# The Relationship between a Competitive School Climate and School Bullying among Secondary Vocational School Students in China: A Moderated Mediation Model

**DOI:** 10.3390/bs14020129

**Published:** 2024-02-10

**Authors:** Xuzhong Huang, Qianyu Li, Yipu Hao, Ni An

**Affiliations:** 1 Normal School of Vocational Techniques, Hubei University of Technology, Wuhan 430068, China; 20201014@hbut.edu.cn (X.H.); 102102401@hbut.edu.cn (Q.L.); anni@whydzscqdlwwgc999.onexmail.com (N.A.); 2 School of Education, Central China Normal University, Wuhan 430079, China

**Keywords:** competitive school climate, school bullying, Chinese secondary vocational students, moderated mediation model, PISA 2018

## Abstract

School bullying is widespread in countries around the world and has a continuous negative impact on the physical and mental health of students. However, few studies have explored the influence mechanism of a competitive school climate on school bullying among Chinese secondary vocational school students. This study aims to explore the relationship between a competitive school climate and bullying in secondary vocational schools in the Chinese context, as well as the mediating role of school belonging and the moderating role of gender. Logit regression analysis and a moderated mediation model were used to analyze 1964 secondary vocational students from China based on PISA 2018 data from Beijing, Shanghai, Zhejiang, and Jiangsu, China. (1) The detection rate of school bullying in secondary vocational schools in China is 17.8%, lower than the world average. (2) A competitive school climate is significantly and positively correlated with secondary vocational school students’ exposure to school bullying. (3) A moderated mediation model suggests that school belonging is an important mechanism by which a competitive school climate influences the occurrence of school bullying, whereas gender moderates the direct effect of a competitive school climate and the indirect effect of school belonging, which mitigates the negative effects of a competitive school climate to some extent. The research results show that creating a healthy competitive climate in schools, cultivating students’ sense of belonging, and facing up to gender differences are helpful to prevent school bullying in secondary vocational schools.

## 1. Introduction

In the existing literature, school bullying is widely regarded as a situation in which one person suffers from prolonged and repeated negative aggressive behavior from one or more people and has difficulty in defending themselves [1]. The phenomenon of school bullying is widespread in education systems around the world [2]. Numerous studies have found that the risk of headache [3], insomnia [4], anxiety [5,6], depression [7,8], and other symptoms in students who suffer from school bullying has increased significantly; these students are prone to skip classes [9], which can cause a decline in academic performance in the short term [10], and in the long term may affect their future employment [11] and life in general [12]. In order to promote the sustainable development of students’ physical and mental health, China has set requirements for the management of school bullying. Since 2019, China has attached great importance to the elevation of the status of vocational education and has continued to increase investment in it. Secondary vocational schools in China have actively explored the construction of a mechanism for preventing school bullying, which has curbed its occurrence to a certain extent. Existing research shows that the overall incidence of school bullying in China has declined significantly and is lower than the world average [13]. However, incidents of school bullying in Chinese vocational education are often reported in media outlets. For example, “a boy at a vocational school in China fell to his death after being bullied twice a day” [14] and “a 16-year-old girl from a secondary vocational school in China was bullied by three other girls” [15]. These incidents have been reported by many media outlets and triggered public outcry. In China, secondary vocational school is a type of education that specializes in technical training and skills to promote employment [16]. It is equivalent to the high school in general education (grades 10–12 of a K-12 school). The age range of secondary vocational school students is generally 15–18 years old [17]. Of China’s policy documents, *Guidance on the Prevention and Treatment of Bullying and School Violence* issued in 2016, mentioned secondary vocational schools when proposing a bullying management program for primary and secondary schools, and that there is no special policy on bullying in vocational education schools. Bullying in vocational schools has not attracted the attention of the Chinese government.

School climate is a key factor reflecting interpersonal relationships among school members, rules and regulations, etc., which can be perceived by students and subconsciously influence their psychology and behavior [18]. Numerous Western studies have shown that a positive and friendly school climate can help improve school bullying, while a competitive school climate may increase the likelihood of bullying [19,20]. Previous studies have shown that a competitive environment is closely related to immoral behavior [21]. Students in a competitive environment tend to be more prone to aggressive behavior. Some teenagers may adopt bullying as a way to emphasize their dominant position when competing with their peers [22]. Teachers and students have also reported that a competitive school climate makes them feel insecure [23]. It can be inferred that a competitive school climate is related to school bullying. The sense of school belonging has also been proven to be related to school bullying in previous studies. Students with a stronger sense of school belonging are less likely to suffer from school bullying [24]. When students take the initiative to integrate into a group and develop a strong connection with the school, it helps them to avoid bullying [25]. Students’ perception of the school and their sense of belonging will also be affected by the school climate [26]. For example, the school climate will affect students’ relationship with their peers, which in turn affects the possibility of being bullied on campus [27]. That is, the sense of school belonging will be affected by the school climate and then affect the bullying behavior of adolescents. School belonging is likely to be an important mediating variable that cannot be ignored. In addition, due to the differences in the innate endowments and acquired cultures of male and female individuals, individuals of different genders have different perceptions of the school climate, and the probability of male and female students suffering from school bullying is also different [28,29]. Therefore, the gender difference in school bullying is also one of the focuses of this study.

In view of this, this paper intends to use PISA 2018 data from Beijing, Shanghai, Zhejiang, and Jiangsu, China, with school belonging as the mediating variable and gender as the moderating variable, to explore the influence mechanism of school climate on school bullying, with a view to promoting China’s secondary vocational school bullying governance and international vocational education school bullying governance. The main contributions of this study are: Firstly, this paper examines the influence mechanism of a competitive school climate on the occurrence of bullying in secondary vocational schools, provides Chinese evidence for the study of bullying in vocational schools, and further deepens the understanding of the climate of bullying in vocational schools. Secondly, this paper helps to improve the climate of school bullying in vocational education, promotes the healthy and sustainable development of secondary vocational students, and provides a theoretical basis and empirical support for the improvement of the climate of secondary vocational schools, which improves the sense of belonging in school and clarifies the gender differences in terms of school bullying.

## 2. Literature Review

### 2.1. School Bullying and the School Climate

The ecological systems theory points out that individual development is the result of the interaction between an individual and the environment [30]. As a part of a microsystem, a school is an important living environment in the process of adolescent growth, which will have a profound impact on students’ psychological state and behavioral development [31]. This study will attempt to explore the specific impact of an important element of the school—the school climate—on school bullying. School climate research had its beginnings in the 1930s, when Kurt Lewin [32] first explored the interaction between individuals and their environment from a psychological perspective, opening the way for research on the effects of the school climate. Hoy and Hannum [18] believed that the school climate unites the characteristics of the school environment, which can be perceived by school members, has an important impact on their psychology and behavior, and has relative stability. According to the symbolic interaction theory, individuals grow and develop in interaction with the environment [33], and students are inevitably affected by the school climate during their interaction with the school. The school climate can be characterized by competition or cooperation [34]. Research has shown that there are obvious differences in the impact of different types of school climate on school bullying [35]. Good teacher–student relationships and solid peer relationships have been associated with students’ perceived positive school climate [19,36], which has a significant effect on the prevention of school bullying. In contrast, a school climate that emphasizes competition may promote school bullying, which students use as a means of coping with intense competition [22]. Competition process is like a game of resource acquisition. In the absence of external constraints, individuals are likely to resort to unethical behaviors (e.g., bullying, violence, etc.) to compete for limited resources [37], which in turn exacerbates the occurrence of school bullying. The theory of social domination also supports this view that students resort to bullying in order to gain higher power and status among their peers [38]. Vocational education, as one of the types of education most closely linked to the labor market [39], and the competitive mechanisms of the labor market have also subtly influenced the school climate of secondary vocational schools for a long time. In other words, a competitive school climate is likely to have some influence on bullying in secondary vocational schools. This paper attempts to dig deeper into the relationship between the two.

### 2.2. The Mediating Role of School Belonging

School belonging mainly refers to the degree of acceptance, respect, and identification that students perceive in school, and can reflect an individual’s emotional connection to the school [40]. A higher sense of school belonging can enable students to have more positive emotions, enhance their attachment to the school [41], help them achieve a positive self-evaluation [42], and promote higher academic performance [43]. School climate is highly correlated with school belonging, and a favorable school climate can help students to have more positive experiences of school, which in turn promotes the formation of students’ sense of belonging to the school [44]. Students with a strong sense of school belonging often have a harmonious peer relationship, have greater trust in teachers, and are more willing to abide by the regulations of the school, which can alleviate the negative impact of a negative school climate to some extent. Studies have shown that school belonging is also significantly negatively correlated with school bullying, and strengthening the sense of belonging to a school has an important preventive and controlling role in reducing bullying behavior among adolescents [45]. A study based on a sample of 6176 Chinese primary school students found that a positive school climate can enhance the sense of school belonging, thereby reducing bullying on campus [46]. A review of studies based on school belonging also found that school belonging was positively correlated with students’ social adaptation, psychological well-being, and self-concept, and negatively correlated with delinquent behaviors such as school bullying [47]. School belonging is likely to mediate the link between school climate and school bullying.

### 2.3. The Moderating Effect of Gender

Gender differences in bullying in schools have received widespread attention from scholars. Studies have shown that the probability of bullying in different genders is not the same. Most of the evidence shows that the bullying rate of boys is higher than that of girls [48]. Boys are more likely to suffer from physical bullying and girls are more likely to suffer from relational bullying [49]. A large-scale survey based on 114,290 primary and secondary school students in 15 provinces and cities in China shows that the incidence of school bullying among boys is higher than that among girls [50]. The Education Longitudinal Study (ELS: 2002) conducted by the National Center for Education Statistics (NCES) in the United States of America shows that students attending co-educational high schools suffer more bullying than those in single-sex high schools, with boys attending co-educational high schools experiencing the highest rates of school bullying [51]. However, there are dissenting voices, with a study based on the 2011–2019 American Youth Risk Behavior Survey showing that girls have a higher incidence of both traditional bullying and cyberbullying than boys [52]. In addition, the results of a survey from Kenya showed that all-boy schools were less likely to report bullying than all-girls or co-educational schools [53]. Evidence from China’s vocational schools shows that although the incidence of bullying among boys is significantly higher than that among girls, the trend is for the proportion of bullying in schools for girls to overtake that of boys [54]. These findings not only reflect gender differences in school bullying across cultures and backgrounds, but also indicate the need to study gender differences in secondary school bullying based in the Chinese context. In addition, perceptions of the school climate and sense of school belonging differed by gender. Under the influence of Chinese traditional culture, boys have stronger self-esteem and higher desire for success [55]. They often hope to show self-confidence and independence through aggression [56], while girls rely more on social interaction with the outside world to meet their emotional needs and are more sensitive to external feedback [57]. That is to say, girls are more susceptible to the influence of the school climate, and boys’ sense of school belonging is significantly lower than that of girls. That means gender is likely to have a role in moderating the effects of school climate and school belonging on school bullying.

### 2.4. PISA and Chinese Vocational School Bullying Research

There has been some research on school bullying using PISA survey data. School bullying in 71 countries based on PISA 2018 found that the problem of school bullying is still prevalent worldwide [58]. Comparing the differences between PISA 2015 and PISA 2018 data, it was found that the problem of school bullying among adolescents in China and Japan has improved significantly, while school bullying among adolescents in the USA and the UK has become more serious; there is no significant change among South Korean adolescents [59], which may be related to the different social cultures, government governance, and school climate in the different countries. The school climate is an important variable that cannot be ignored when studying school bullying. Based on the PISA 2018 data, a comparison of 64 countries shows that a cooperative school climate plays a protective role in school bullying [60]. Cross-cultural research on Chilean and South African students also provides evidence that a positive school climate can reduce school bullying [61]. In addition, studies based on PISA data from the United States, Australia, and other countries have indicated that school belonging is correlated with school bullying, and school belonging can mitigate the negative effects of school bullying to a certain extent [62,63]. Chinese studies using PISA data have also addressed the relationship between school climate, school belonging, and school bullying. The results show that, in the context of Chinese schools, a positive school climate helps to reduce school bullying [64], and school bullying has a weakening effect on school belonging [65]. However, the relevant research is either aimed at general education students or focuses on a cooperative school climate; there is no direct research on the impact of a competitive school climate on the bullying of secondary vocational students. From an international perspective, existing research based on the PISA data is relatively comprehensive, but there is a lack of research on Chinese vocational school students. The existing research on the sample of Chinese vocational education students only focuses on the current situation of school bullying [66]; empirical studies have not been conducted on the correlation mechanism between a competitive school climate and school bullying.

**Figure 1:**
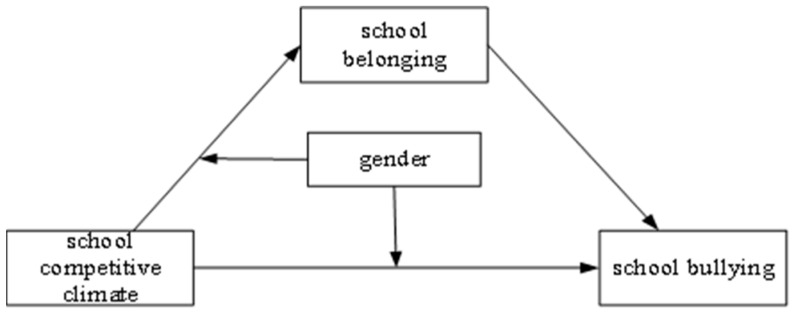
Hypothesized model of the effect of school competitive climate on school bullying.

Therefore, this paper attempts to use PISA 2018 data from Beijing, Shanghai, Zhejiang, and Jiangsu in China to build on the initial analysis of the impact of a competitive school climate on school bullying. We will complete this by using a moderated mediation model to analyze the mediating mechanism played by school belonging in the impact of a competitive school climate on school bullying and the moderating role of gender (Figure 1). We aim to clarify the specific influence mechanism of a competitive school climate on school bullying, and to empirically explore effective ways to solve the problem of school bullying in vocational schools in China.

## 3. Methods

### 3.1. Data and Samples

The present study used data from PISA 2018 by the Organisation for Economic Cooperation and Development (OECD). It is an international academic achievement test that collects data on students’ cognitive and non-cognitive skills, as well as their family backgrounds and school environments, making it comprehensive and globally relevant. In 2018, 12,058 students from 361 schools in Beijing, Shanghai, Zhejiang, and Jiangsu provinces and cities in China participated in the PISA test, of which 9901 students were enrolled in general secondary schools and 2157 students were enrolled in secondary vocational schools. Considering the need for research modeling, this study used the column-censoring method to eliminate samples with missing values, and finally obtained a sample of 1964 Chinese secondary vocational school students.

### 3.2. Models and Variables

#### 3.2.1. Variables

- School bullying

PISA 2018 adopts a self-assessment questionnaire from the perspective of the bullied person. The school bullying research in this paper is also from the perspective of the bullied person, that is, whether or not a student suffers from school bullying. Students chose between the options “Never or almost never,” “A few times a year,” “A few times a month,” and “Once a week or more”, according to descriptions of six behaviors: (1) “Other students left me out of things on purpose”; (2) “Other students made fun of me”; (3) “I was threatened by other students”; (4) “Other students took away or destroyed things that belonged to me”; (5) “I got hit or pushed around by other students”; (6) “Other students spread nasty rumors about me”, based on their own experience in the last 12 months. Referring to existing research [67], students who said that they had experienced one or more of these bullying behaviors “A few times a month” or “Once a week or more” in the past 12 months were defined as suffering from school bullying and assigned a value of 1, while the remaining cases were defined as not suffering from school bullying, with a value of 0. We defined topics 1 and 6 as relational bullying, topics 2 and 3 as verbal bullying, and topics 4 and 5 as physical bullying. Students who have suffered at least one verbal, relational, or physical bullying behavior were defined, respectively, as students who have suffered verbal bullying (assigned a value of 1), students who have suffered relational bullying (assigned a value of 1) and students who have suffered physical bullying (assigned a value of 1), and those not experiencing any of these behaviors were assigned a value of 0.

- Competitive school climate

School climate is mainly derived from students’ perceptual experience of school life. Based on the PISA 2018 data framework, this study used students’ perceptions of the climate in their school as an important indicator of a competitive school climate. Specifically, the competitive school climate was examined in relation to four questions: (1) Students seem to value competition; (2) It seems that students are competing with each other; (3) Students seem to share the feeling that competing with each other is important; (4) Students feel that they are being compared with others. Scores were assigned, ranging from “Not at all true” (1) to “Extremely true” (4), for each option. The item scores were then transformed using Item Response Theory (IRT), and the transformed variables were analyzed.

- School belonging

School belonging is based on students’ responses to six statements: (1) “I feel like an outsider (or left out of things) at school”; (2) “I make friends easily at school”; (3) “I feel like I belong at school”; (4) “I feel awkward and out of place in my school”; (5) “Other students seem to like me”; and (6) “I feel lonely at school.” The answers to questions 2, 3, and 5 were assigned the value of 1–4 from “Strongly Disagree” to “Strongly Agree,” and the answers to questions 1, 4, and 6 were scored in reverse; this was then transformed into a school belonging index by using the Item Response Theory (IRT), in which higher scores indicate a higher sense of belonging to the school.

- Control variable

Referring to the ecosystem theory and existing literature, we selected individual characteristics, family factors, and school factors that have an impact on students as control variables in the measurement model. Specifically, individual student characteristics included gender (in “Are you female or male?” the answer “female” is scored as 1, and the answer “male” is scored as 0), learning literacy (according to the Plausible value of mathematics, science, and reading provided by PISA, the first Plausible value is used as a proxy variable), non-cognitive ability (the synthesis of four dimensions: positive emotions, resilience, achievement motivation, and self-efficacy), the self-education expectation (for “Which of the following do you expect to complete?” the value of the answer to “ISCED level 5B” or “ISCED level 5A or 6” is 1, and remaining values are 0), life satisfaction (continuous variables are synthesized by students’ scoring), ESCS (the IRT technique was applied to synthesize the data through the variables of parents’ highest level of education, parents’ highest occupational status, and household wealth) and parental emotional support (based on the answers to “My parents support my educational efforts and achievements”; “My parents support me when I am facing difficulties at school”; “My parents encourage me to be confident”; a continuous variable was synthesized by IRT analysis). The school characteristics variables included teacher support (the answers to “The teacher shows an interest in every student’s learning”; “The teacher gives extra help when students need it”; “The teacher helps students with their learning” are synthesized into continuous variables by IRT analysis), teacher qualifications (the manager’s questionnaire is weighted by the number of master’s degrees and above to synthesize continuous variables), school location (the value of the answer to “your school area” in the manager’s questionnaire is 1 for ‘a city’ or ‘a large city’, and 0 for the rest), school nature (in the manager questionnaire “Is your school a public or a private school?” the answer “public school” is scored as 1, and a private school is scored as 0), and cooperative school climate (a continuous variable was synthesized from the responses to the four statements “Students seem to value cooperation”; “It seems that students are cooperating with each other”; “Students seem to share the feeling that cooperating with each other is important”; “Students feel that they are encouraged to cooperate with others”, analyzed by IRT).

#### 3.2.2. Models

We used Stata 16.0 statistical software and PROCESS4.0 for data processing and analysis. The tests were considered statistically significant if the two-sided *p*-value was less than 0.05. The data analysis of this study was mainly divided into three stages.

First, we used Stata 16.0 to sort out, convert, and process the missing values of Chinese data. The statistical analysis was weighted according to the student weight (W_FSTUWT) and school weight (W_SCHGRNRABWT) provided by PISA 2018 to obtain the data of Chinese secondary vocational students. Then, we used descriptive analysis to understand the possibility of bullying among secondary vocational school students in China, and compared it with the level of bullying among ordinary high school students in China and in other countries around the world, so as to present the overall prevalence of bullying among secondary vocational school students in China.

Secondly, on the basis of the descriptive analysis, we took school bullying variables as dependent variables, a competitive school climate as the independent variable, and students, families, teachers, schools, and other related variables as control variables to establish a statistical analysis model.

The specific model had the following form:

$$\ln\left(\frac{P_{bully_{i}=1}}{1-P_{bully_{i}=1}}\right)=\alpha +\beta_{1}X_{1}+\beta_{2}X_{2}+\ldots \beta_{n}X_{n}+\varepsilon$$

where i refers to the i-th secondary vocational school student, $bully_{i}=1$ means that the i-th student said they had suffered from school bullying, $P_{bully_{i}=1}$ is the probability that a student suffered from school bullying, α is a constant, $X_{n}$ is an explanatory variable including a competitive school climate, $\beta_{n}$ is the related coefficient of variation, and ε is the model error term. In terms of data results, this paper directly presents the occurrence ratio results of a Logit regression.

Finally, a PROCESS4.0 plug-in was used in SPSS25 to analyze the moderated mediation model, verify the mediating role of school belonging and the moderating role of gender, and empirically explore the influence mechanism of a competitive school climate on school bullying of secondary vocational school students.

## 4. Results

### 4.1. Descriptive Statistic

Table 1 presents the results of descriptive statistics for all variables. The results show that the proportion of secondary vocational school students who have suffered from school bullying in China is 17.8%, which indicates that the problem should not be underestimated. The comprehensive score of a competitive school climate is −0.041 points. Students who had suffered from school bullying had significantly higher competitive school climate scores than those who had not suffered from school bullying. This suggests that a competitive school climate is likely to be strongly associated with the occurrence of school bullying, and it is urgent to verify this with further research.

### 4.2. School Bullying among Secondary School Students in China

**Figure 2:**
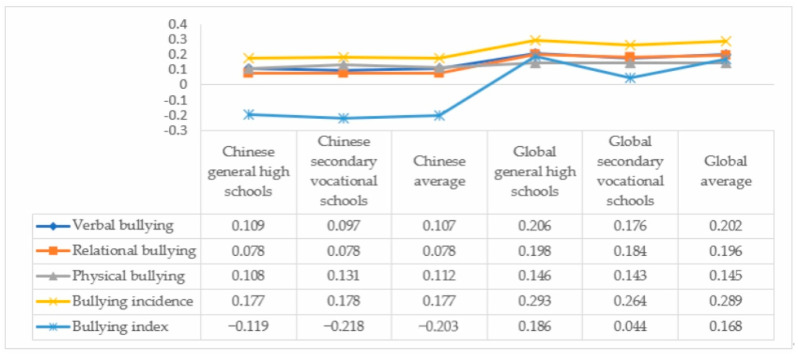
PISA2018 school bullying status results.

Figure 2 shows that there is no significant difference in school bullying among different types of education. School bullying in secondary vocational schools in China shows a basic trend of (decreasing sequentially) physical–verbal–relational. Specifically, first, in terms of international comparisons, China’s general high school, secondary vocational school, and average bullying rates are lower than those of other OECD countries. Second, from the perspective of internal comparison, there is no significant gap between the bullying rate of secondary vocational schools and general high schools in China, and there are only differences in specific manifestations. Third, in terms of specific bullying behaviors, the bullying situation of secondary vocational school students showed a basic trend of physical bullying (13.1%) > verbal bullying (9.7%) > relational bullying (7.8%).

### 4.3. The Effect of Competitive School Climate on School Bullying

Table 2 displays the results of the effects of a competitive school climate and other control variables on school bullying among Chinese secondary vocational school students, where the dependent variables for Models 1–4 are the incidence of all school bullying, verbal bullying, relational bullying, and physical bullying. In addition, the student bullying index created by PISA has been empirically supported by samples from multiple countries. In this study, the bullying index is used as the dependent variable in Model 5 to test the robustness. The results showed that the variables of learning literacy, the self-education expectation, ESCS, parental emotional support, teacher qualifications, school location, and school nature had a non-significant effect, whereas gender, non-cognitive ability, life satisfaction, teacher support, and cooperative school climate had a significant negative effect on students suffering from school bullying. Using the bullying index to replace the dependent variable for analysis, it was found that the competitive school climate can still significantly positively predict the occurrence of school bullying. From the core independent variables of this paper, and controlling for the effects of other variables, the competitive school climate perceived by Chinese secondary vocational school students had a significantly positive predictive effect on their suffering from school bullying (school bullying incidence = 1.566, *p* < 0.001). Further analyses of the specific effects of a competitive school climate on secondary vocational students suffering from different types of school bullying found that a competitive school climate had a significantly positive effect on verbal bullying, relational bullying, and physical bullying (verbal bullying incidence = 1.497, *p* < 0.001; relational bullying incidence = 1.734, *p* < 0.001; physical bullying incidence = 1.566, *p* < 0.001).

### 4.4. A Moderated Mediation Model

This section uses Model 8 in the SPSS macro program prepared by Hayes to test for mediation and moderation effects. After controlling for other variables, the results of the regression analysis are shown in Table 3 through Bootstrap 5000 samples sampled with a 95% confidence interval as the evaluation standard. The results of a Harman single factor experiment showed that the variation in the first factor extracted by the core variable measurement item was 18.53%, which was lower than the standard of 40%; that is, there was no obvious problem of common method bias. From the stepwise regression results, we see that a competitive school climate significantly predicted school belonging and school bullying, respectively. With the inclusion of both competitive school climate and school belonging in Model 3, the direct effect of a competitive school climate on school bullying was reduced but still significant. The direct effect of school belonging on school bullying was equally significant. The results of the mediation effect test showed a 95% confidence interval of [0.0205, 0.0870], which did not contain 0, indicating that the mediation effect of school belonging was significant.

In order to further test the moderating effect of the gender variable, referring to existing research [68], the direct and indirect effects of the moderating effects were tested at different levels of the gender variable. As shown in Table 4, in terms of direct effects, the direct effect of a competitive school climate on school bullying was significant for both boys and girls, but the direct effect was greater for girls. The indirect effect of a competitive school climate affecting school bullying among male students through school belonging was 0.0520, with a confidence interval of [0.0199, 0.0960], and its indirect effect was significant, whereas the indirect effect on female students was 0.0074 and its indirect effect was not significant. Further calculation of the mediation effect judgement indicator with moderation gave an INDEX value of −0.0446, and the 95% confidence interval was [−0.1042, −0.002], which excluded 0.

## 5. Discussion

Based on the PISA 2018 survey data, this paper used the logit regression model and the moderated mediation model to explore the situation of school bullying among secondary vocational students in China. A systematic assessment of the actual impact of a competitive school climate on school bullying and its mechanisms was carried out, the specific pathway of “competitive school climate–school belonging–school bullying” was identified, and the moderating role of gender in this pathway was verified. The specific findings of the study are set out below:

First, compared with the report released by the United Nations Educational, Scientific and Cultural Organization (UNESCO) in 2019, in which the global incidence of school bullying is about 31% [69], the overall incidence of school bullying in China has been significantly lower than the global average. From the perspective of Chinese secondary vocational school students, the bullying rate is 17.8%, which is still significantly lower than the world average. It can be seen that China’s school bullying governance has achieved certain results. A comparative study of Chinese and American students has found that Chinese students have a more positive view of the school climate [70]; perhaps this is because China advocates fairness, adaptability, and healthy competition. According to economic theory, when team members are in fair competition, they perceive the matching of input and output, which makes it easier for them to generate positive intrinsic motivation and implement positive behavior [71]. In this study, it is also pointed out that the score of competitive school climate perceived by Chinese secondary vocational students is −0.041, which is lower than the world average. This may also be related to the emphasis on harmonious education in Chinese traditional culture [72]. China emphasizes harmonious education and is committed to creating a fair, standardized, and disciplined school climate, which may provide a reference for school bullying governance in other countries around the world.

Second, a competitive school climate has a significantly positive predictive effect on school bullying among secondary vocational students. For every additional unit of competitive school climate, the possibility of secondary vocational students in China suffering from school bullying increases by 56.6%, which is basically consistent with previous research conclusions [73,74]. The results reveal that, in the Chinese context, if the competitive school climate of secondary vocational schools is stronger, there will be greater likelihood that students will suffer from school bullying. In addition, the more competitive the school climate is perceived to be by secondary vocational students, the higher the possibility that students will suffer from verbal bullying, relational bullying, and physical bullying, among which the possibility of relational bullying is the highest. This finding is basically consistent with the conclusions of the existing literature on the field of general education [75]. That is, whether in the field of general education or vocational education, a competitive school climate will contribute to the occurrence of school bullying to a certain extent. Governments and schools should pay attention to the negative impact of the competitive school climate, and not blindly encourage competition. School administrators should formulate more fair, just, and open school rules and regulations to ensure that the opportunities, processes, and results of competition are open and transparent, and reduce the negative impact of an unfair and vicious climate.

Third, the research findings indicate that school belonging plays an intermediary role in the competitive school climate and school bullying. On the one hand, the more competitive a climate that students perceive, the lower their sense of school belonging and the higher their likelihood of being bullied. The mediating effect of school belonging might be due to the fact that a strongly competitive school climate leads students to compete with each other for limited resources, making it difficult for them to form recognition and feel a sense of belonging to the school, and thus exacerbating the possibility of students suffering from school bullying. Organizational research shows that competitive relationships within the organization will induce individuals to act in their own self-interest, increase behaviors that are detrimental to others, interfere with the sense of team trust and group cohesion [76], and consequently reduce secondary vocational school students’ sense of belonging to the school. On the other hand, school belonging can alleviate the positive predictive effect of a competitive school climate on school bullying. School belonging is often related to higher levels of well-being and adaptability, and is one of the protective factors of school bullying [77]. In this regard, schools can encourage students to integrate into the school as ‘masters’, put forward students’ perspectives for the construction of school culture, and create a good campus life experience for students by providing students with more abundant and diverse resources to meet their individual needs, so as to increase students’ sense of identity and participation in the school. Secondly, teachers should give students positive emotional support, help students establish correct self-worth cognition, and guide students toward non-violent means to solve friction with their peers, so as to cultivate students’ sense of school belonging by enhancing their trust in teachers [78].

Fourth, gender plays an important moderating role in the direct effect of a competitive school climate on school bullying and the indirect effect of school belonging. The results reveal that, compared with female students, male students are more adaptable to the competitive climate of secondary vocational schools and thus more likely to have a sense of school belonging, which may stem from the differences in self-identity of the different genders [79]. In the context of Chinese society, boys are mostly raised to be “men” who demonstrate their self-worth through independence; therefore, they can better adapt to a competitive school environment. However, girls often show submissive and obedient qualities in school [80], and pay more attention to interpersonal relationships [81]. They often obtain recognition through outward socializing and maintaining peer relationships, which is easily affected by the external environment. A strongly competitive climate is not conducive to girls maintaining good social relationships, and even less conducive to their sense of school belonging. In this regard, parents and teachers should guide boys to adopt appropriate and non-violent forms of benign competition. At the same time, teachers should devote more time, energy, and resources to girls, helping them to actively participate in school activities, so as to alleviate the negative impact of the competitive school climate on girls.

## 6. Implications

Based on the research conclusions, it is necessary to improve the school climate to reduce the occurrence of school bullying. Studies have shown that an overly competitive school climate will increase the incidence of adolescent problem behaviors. This study provides empirical evidence from China to support this conclusion. At the same time, this study supplements the research on the competitive climate of schools. Students are more likely to show aggressive behavior when they perceive a strongly competitive climate, which leads to school bullying. School bullying in a competitive climate can be seen as an aggressive means to obtain scarce resources or achieve certain personal goals [82], and enhancing students’ sense of school belonging can reduce the negative impact of the competitive climate. Reducing the competition in society and schools, creating a fair and orderly benign competition atmosphere, and guiding students to view competition with a healthier mindset will help to reduce bullying among secondary vocational students. Educators should focus on providing emotional support for students, establishing good teacher–student relationships, and helping students establish friendly peer relationships to enhance the sense of school belonging.

## 7. Limitations and Future Research

This study has the following limitations. First, as it is limited by the data, this paper may also have the problem of missing variables. For example, PISA data do not measure students’ personality traits, and personality traits are also an important factor affecting the occurrence of school bullying [83]. Secondly, this paper used the column deletion method to delete data with missing value samples, and the proportion of deleted samples was about 8.9%. This rough processing method will inevitably affect the accuracy of the estimation results. Thirdly, PISA 2018 only considered data from Beijing, Shanghai, Zhejiang, and Jiangsu, but these four provinces and cities are located in China’s coastal areas and have a high level of economic development. The research conclusions cannot be extended to all of China, and quantitative research cannot fully assess how a competitive campus atmosphere affects the occurrence of campus bullying. Finally, this study used self-report questionnaires as the main source of information. The competitive school climate and school belonging were reported on by students and are subjectively influenced by students. Future research can employ the nomination method to acquire peer assessment data for further in-depth investigation.

## 8. Conclusions

The findings of this study, based on a sample of secondary vocational school students from four Chinese provinces and cities in PISA 2018, suggest that there is a significantly positive predictive effect of competitive school climate on Chinese secondary vocational school students suffering from school bullying, while school belonging mediates the relationship between the two. These findings are of great significance. First, the problem of school bullying in China’s secondary vocational education needs more attention. Second, a competitive school climate has a significant negative impact on school bullying in Chinese secondary vocational schools. Third, school belonging mediates school climate and school bullying in the Chinese context. These findings complement and extend the research on the association between school bullying and school climate and also explore the mediating role of school belonging, which is of great significance to the study of school bullying in China’s secondary vocational education.

## Figures and Tables

**Table 1:** Results of descriptive statistical analysis of variables.

Variable	Full-Sample		Bullied		Not Bullied	
	M	SD	M	SD	M	SD
School bullying	0.178	0.381	1	0	0	0
Competitive school climate	−0.041	0.913	0.172	0.934	−0.087	0.902
Gender	0.442	0.497	0.313	0.464	0.470	0.499
Learning literacy	0	1	−0.079	1.052	0.017	0.988
Non-cognitive ability	2.970	0.327	2.885	0.324	2.988	0.324
The self-education expectation	0.269	0.444	0.249	0.433	0.274	0.446
Life satisfaction	6.932	2.424	5.983	2.685	7.135	2.316
ESCS	−0.813	0.946	−0.885	0.943	−0.798	0.946
Parental emotional support	−0.173	0.864	−0.296	0.876	−0.147	0.859
Teacher support	0.338	0.956	0.060	1.030	0.398	0.929
Teacher qualifications	0.166	0.164	0.160	0.158	0.167	0.166
School location	0.780	0.415	0.748	0.435	0.786	0.410
School nature	0.930	0.255	0.913	0.282	0.934	0.249
Cooperative school climate	0.073	0.994	−0.101	1.017	0.110	0.986

**Table 2:** Results of Logit regression model.

Variable	Model 1	Model 2	Model 3	Model 4	Model 5
	School Bullying	Verbal Bullying	Relational Bullying	Physical Bullying	Bullying Index
Competitive school climate	1.566 ***	1.497 ***	1.734 ***	1.566 ***	0.136 ***
	(0.115)	(0.136)	(0.171)	(0.129)	(0.026)
Gender	0.489 ***	0.315 ***	0.408 ***	0.485 ***	−0.362 ***
	(0.064)	(0.058)	(0.078)	(0.074)	(0.045)
Learning literacy	0.999	0.998	0.996 **	0.998	−0.001 **
	(0.001)	(0.001)	(0.001)	(0.001)	(0.000)
Non-cognitive ability	0.552 **	0.694	0.720	0.700	−0.134
	(0.126)	(0.196)	(0.221)	(0.180)	(0.085)
The self-education expectation	1.118	1.034	1.153	1.187	0.059
	(0.174)	(0.207)	(0.249)	(0.209)	(0.054)
Life satisfaction	0.872 ***	0.838 ***	0.826 ***	0.861 ***	−0.081 ***
	(0.023)	(0.026)	(0.028)	(0.025)	(0.010)
ESCS	0.984	0.982	1.033	0.955	−0.015
	(0.070)	(0.086)	(0.099)	(0.077)	(0.026)
Parental emotional support	0.971	0.964	0.908	1.004	−0.018
	(0.080)	(0.099)	(0.102)	(0.093)	(0.029)
Teacher support	0.805 ***	0.853 *	0.735 ***	0.822 **	−0.110 ***
	(0.051)	(0.067)	(0.061)	(0.059)	(0.025)
Teacher qualifications	1.178	0.691	1.294	1.134	0.044
	(0.501)	(0.385)	(0.741)	(0.552)	(0.151)
School location	0.865	1.135	1.497	0.837	0.010
	(0.134)	(0.227)	(0.353)	(0.147)	(0.058)
School nature	0.974	1.386	1.055	0.832	−0.019
	(0.237)	(0.431)	(0.339)	(0.220)	0.096
Cooperative school climate	0.857 **	0.855	0.864	0.924	−0.046
	(0.061)	(0.075)	(0.082)	(0.074)	(0.026)
*N*	1964	1964	1964	1964	1547

Robust standard errors for incidence ratios are in parentheses; *, **, and *** are significant at the 0.05, 0.01, and 0.001 levels, respectively.

**Table 3:** Results of the mediation model.

Variable	Model 1: School Bullying		Model 2: School Belonging		Model 3: School Bullying	
	β	Z	β	t	β	Z
Competitive school climate	1.060 ***	0.011	−0.0763 ***	1.16	1.056 ***	0.011
School belonging					0.949 ***	0.012
Control variable	control		control		control	
School fixed effect	control		control		control	

*** is significant at the 0.001 level; The control variables are the same as in the previous section.

**Table 4:** Results of Bootstrap test for mediated effects with moderation.

		Effect	SD	BootLLCI	BootULCI
Direct effect of competitive school climate	male	0.2558	0.0784	0.1022	0.4095
	female	0.5411	0.1136	0.3184	0.7639
Indirect effect of school belonging	male	0.0520	0.0195	0.0199	0.0960
	female	0.0074	0.0179	−0.0279	0.0441
Mediated mediating effect		−0.0446	0.0262	−0.1042	−0.002

## Data Availability

The data presented in this study are available on request from the corresponding author.
